# Gene expression profiling of chronic myeloid leukemia with variant t(9;22) reveals a different signature from cases with classic translocation

**DOI:** 10.1186/1476-4598-12-36

**Published:** 2013-05-04

**Authors:** Francesco Albano, Antonella Zagaria, Luisa Anelli, Nicoletta Coccaro, Luciana Impera, Crescenzio Francesco Minervini, Angela Minervini, Antonella Russo Rossi, Giuseppina Tota, Paola Casieri, Giorgina Specchia

**Affiliations:** 1 Department of Emergency and Organ Transplantation (D.E.T.O.), Hematology Section - University of Bari, 70124, Bari, Italy

**Keywords:** Chronic myeloid leukemia, Variant t(9;22) rearrangements, Gene expression profiling, Protein kinases, Cellular pathways

## Abstract

**Background:**

The t(9;22)(q34;q11) generating the *BCR/ABL1* fusion gene represents the cytogenetic hallmark of chronic myeloid leukemia (CML). About 5–10% of CML cases show variant translocations with the involvement of other chromosomes in addition to chromosomes 9 and 22. The molecular bases of biological differences between CML patients with classic and variant t(9;22) have never been clarified.

**Findings:**

In this study, we performed gene expression microarray analysis to compare CML patients bearing variant rearrangements and those with classic t(9;22)(q34;q11). We identified 59 differentially expressed genes significantly associated with the two analyzed groups. The role of specific candidate genes such as *TRIB1* (tribbles homolog 1), *PTK2B* (protein tyrosine kinase 2 beta), and *C5AR1* (complement component 5a receptor 1) is discussed.

**Conclusions:**

Our results reveal that in CML cases with variant t(9;22) there is an enhancement of the MAPK pathway deregulation and show that kinases are a common target of molecular alterations in hematological disorders.

## Background

Chronic myeloid leukemia (CML) is a myeloproliferative disorder derived from hematopoietic stem cell transformation and characterized by heterogeneous biological and clinical features. The CML molecular marker is *BCR/ABL1* fusion gene generation as a consequence of a reciprocal t(9;22)(q34;q11) [1,2]. In most cases, the Philadelphia (Ph) chromosome is cytogenetically detectable but about 5–10% of CML patients show variant t(9;22)(q34;q11) rearrangements with the involvement of additional chromosomes [3,4]. In these cases the *BCR/ABL1* fusion gene can be revealed by Fluorescence in situ hybridization (FISH) or reverse transcriptase-polymerase chain reaction [5]. The occurrence of genomic microdeletions proximally to *ABL1* or distally to *BCR* has been reported in CML cases with variant translocations with a greater frequency (30-40%) than in cases with classic t(9;22) (10-18%) [6,7]. The prognostic significance of variant t(9;22) was unclear and debated in the pre-imatinib era, whereas recent studies of large CML series have reported that the presence of variant translocations has no impact on the cytogenetic and molecular response or on prognosis [6,8]. However, the molecular bases of biological differences between CML patients with classic and variant t(9;22) have never been elucidated.

In this study, we performed gene expression profiling (GEP) by microarrays to identify a signature discriminating CML patients bearing variant rearrangements from those with classic t(9;22)(q34;q11). A list of 59 genes was found to be significantly associated with the two analyzed groups showing a differential expression. We applied network analysis to evaluate potential pathways involved in CML heterogeneity. An overall deregulation of genes encoding for protein kinases and involved in crucial cellular pathways such as MAPK (mitogen-activated protein kinase) signaling was found, unveiling the biological basis of differences in the CML patients subgroup with variant rearrangements.

## Findings

**Figure 1:**
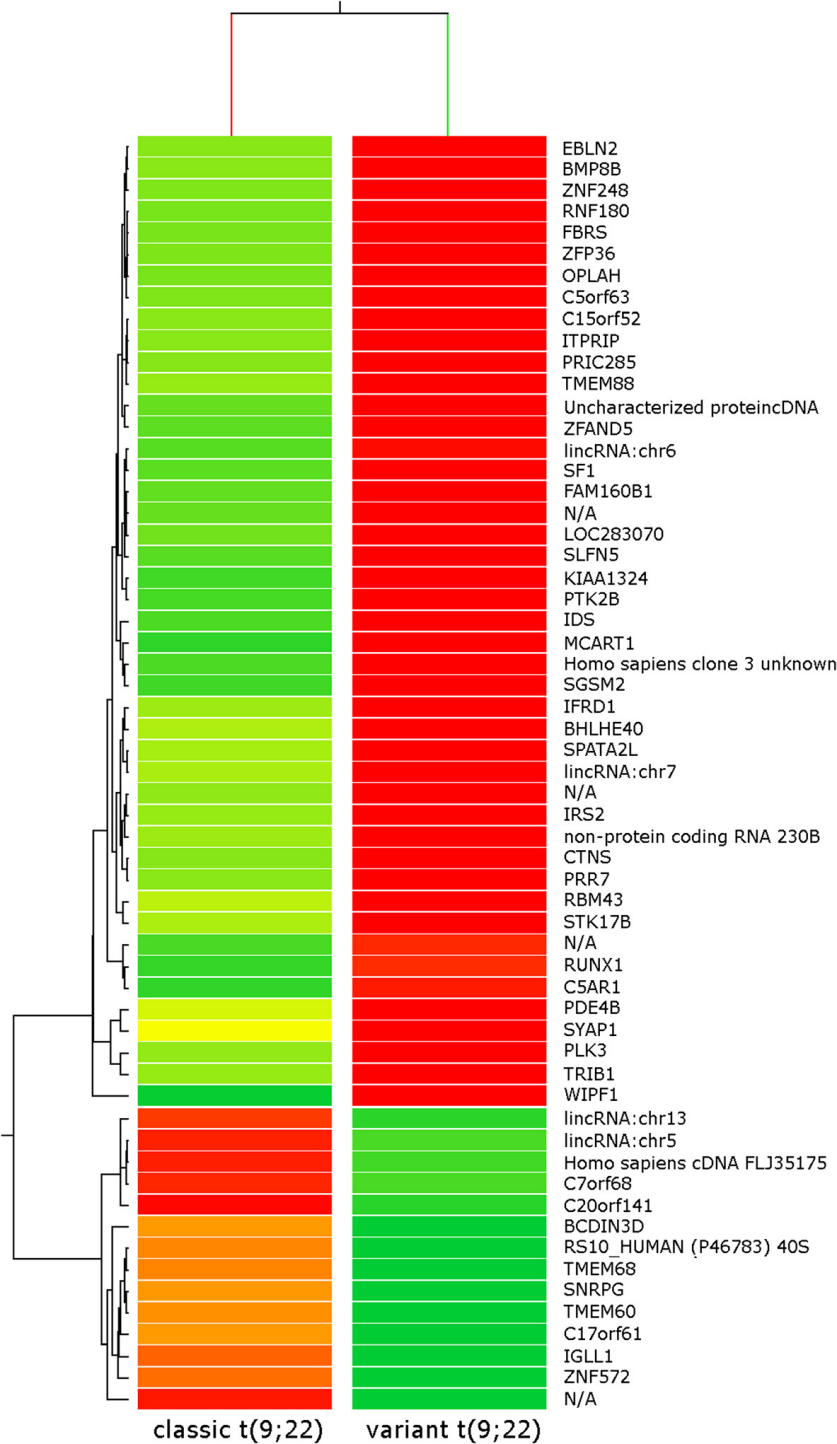
**Hierarchical genes clustering in CML with classic (12 cases) and variant (8 cases) t(9;22) showing a 59-gene signature.** Each row represents a single gene; green indicates differentially decreased expression of each gene in CML patient samples with variant t(9;22) compared with classic t(9;22) and red indicates differentially increased expression. The more saturated the color, the greater the degree of differential expression. N/A indicates genomic sequences not yet annotated.

Banding and molecular cytogenetic analyses allowed the identifications of 12 CML cases with classic t(9;22) and 8 cases with variant translocations (Additional file 1, Additional file 2). The *BCR/ABL*1 fusion analysis revealed the occurrence of b2a2 or b3a2 junctions in 10 (7 with classic and 3 with variant rearrangement) and in 10 (5 with classic and 5 with variant rearrangement) cases, respectively. All these patients were selected for further GEP analysis by oligonucleotide microarrays (Additional file 2). A set of 59 genes was identified as differently expressed in CML cases with variant t(9;22) rearrangements. All deregulated genes showed a more than 2 fold expression change; results of gene expression analysis indicated that 45 out of 59 differentially expressed genes were up-regulated whereas 14 were down-regulated (Figure 1; Additional file 3).

### Upregulation of kinases genes

Querying the Database for Annotation, Visualization and Integrated Discovery (DAVID) showed that the enhanced biological process in our gene set involved the intracellular protein kinases cascade, a series of reactions in which a signal is passed within the cell by sequential protein phosphorylation. This enhancement was significant (p = 0.003) and associated to a value of 7.6. In detail, the kinases list included 5 genes: *TRIB1* (tribbles homolog 1), *STK17B* (serine/threonine kinase 17b), *PTK2B* (PTK2B protein tyrosine kinase 2 beta), *C5AR1* (complement component 5a receptor 1) and *ZFP36* (zinc finger protein 36, C3H type, homolog). Interestingly, *TRIB1* resulted one of the highest expressed genes showing a 2.9-fold change in GEP experiments. The upregulation of all 5 kinases was confirmed by quantitative real-time polymerase chain reaction experiments (qRT-PCR) analysis, with statistically significant expression levels ranging from 2.20 to 8.02.

### Involvement of kinases in the RAS/MAPK pathway

**Figure 2:**
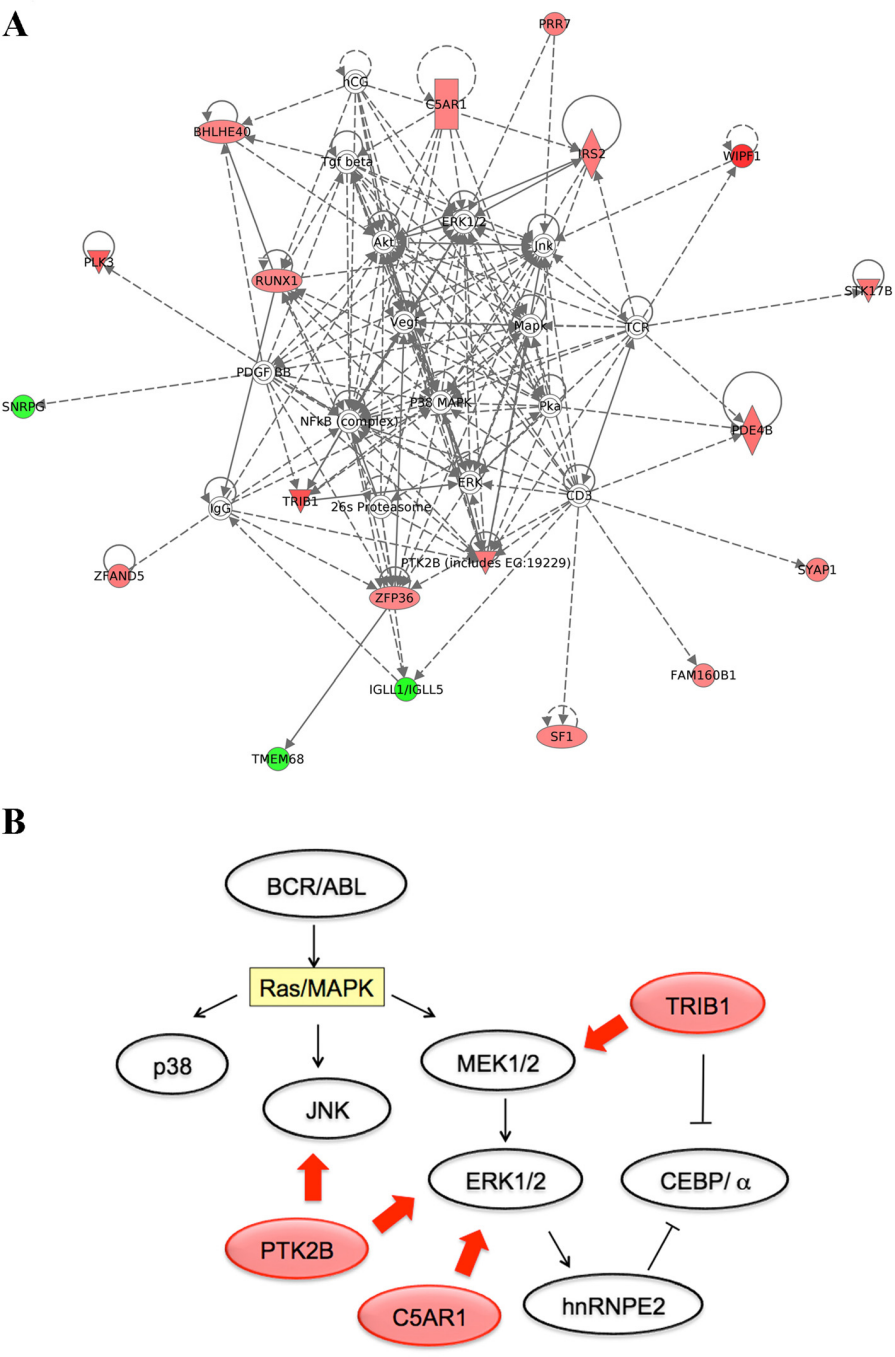
**Deregulated genes in CML cases with variant t(9;22).** (**A**) “Hematological System Development and Function, Tissue Morphology, Cellular Development” network deriving from GEP in CML cases with variant t(9;22). Both direct (solid lines) and indirect (dashed lines) interactions among genes are shown. Colored symbols correspond to genes included in our set of differentially expressed genes (red = upregulated and green = downregulated). (**B**) The involvement of TRIB1, PTK2B and C5AR1 kinases in the RAS/MAPK pathway downstream to the BCR/ABL oncoprotein.

Further Ingenuity Pathways Analysis (IPA) analysis yielded strong indications that 19 out of 59 dysregulated genes from our dataset are involved in the “Haematological System Development and Function, Tissue Morphology, Cellular Development” network (Figure 2A). A central role in this network is played by several proteins that are known to be activated in *BCR/ABL1* cells, namely ERK1/2 (extracellular signal-regulated kinases), p38MAPK (p38 mitogen-activated protein kinase)*,* JNK (c-Jun N-terminal kinase), and cell cycle regulator AKT *(*RAC-alpha serine/threonine-protein kinase) that have a key function in multiple cellular processes such as apoptosis and cell proliferation (Figure 2A). In this respect, three main cellular processes are dysregulated by the BCR/ABL oncoprotein: RAS/MAPK which induces activation of proliferation, the PI3K (phosphatidylinositol-3 kinase)/AKT that activates apoptosis, and JAK/STAT which leads to an increased transcriptional activity [9]. Noteworthy, the upregulated kinase genes, previously revealed by DAVID analysis, are also enclosed in the network identified by IPA and establish direct or indirect interactions with other network components (Figure 2A). Moreover, TRIB1, PTK2B and C5AR1 kinases are involved in the regulation of the RAS/MAPK pathway (Figure 2B) [10-12]. TRIB1 is a signaling regulatory protein involved in leukemogenesis by regulating cellular proliferation and myeloid differentiation [10]. This protein binds to the ‘middle layer’ of kinases in the MAPK network, MAPKK (mitogen activated protein kinase kinase), and acts as an adaptor between the MAPKK pathway and C⁄EBPα (CCAAT⁄enhancer binding protein alpha). In fact, TRIB1 interacts with MEK1 (mitogen-activated ERK kinase 1) and MKK4 (MAP kinase kinase 4) and enhances phosphorylation of ERK1⁄2, promoting cell proliferation and suppressing apoptosis. ERK1/2 phosphorylation is required for C⁄EBPα degradation and the activation of hnRNP-E2 (poly(rC) binding protein 2), a C⁄EBPα repressor (Figure 2B) [10]. Moreover, PTK2B is a proline–rich kinase involved in calcium induced regulation of ion channel and activation of the MAPK signaling pathway by stimulating JNK and ERK1/2 activity (Figure 2B) [11]. PTK2B is a member of the FAK tyrosine kinases family that could be activated by BCR-ABL causing an aberrant cell adhesion. C5AR1 is a member of the rhodopsin family of G protein-coupled receptors and is also the receptor of C5a anaphylatoxin, a strong proinflammatory mediator. It has been recently demonstrated that C5a binding to C5aR on human intestinal epithelial cells activates ERK1/2 and AKT phosphorylation [12].

To date several profiling studies in CML have been reported focusing mainly on predicting response to imatinib therapy and identifying a gene-specific signature for different stages of the disease [13,14]. Our GEP analysis performed on CML cases with variant t(9;22) may improve the understanding of the biological mechanisms at the basis of the CML heterogeneity. Overall, our results reveal that in CML cases with variant t(9;22) there is an enhancement of the MAPK pathway deregulation already known to underlie the CML pathogenesis and point out the role of interesting candidate genes, such as *TRIB1, PTK2B,* and *C5AR1*. These findings show that kinases are a common target of molecular alterations in hematological disorders and reinforce the idea that a perturbed action of signal transduction pathways is one of the hallmarks of cancer.

## Abbreviations

CML: Chronic myeloid leukemia; Ph: Philadelphia chromosome; FISH: Fluorescence In Situ Hybridization; GEP: Gene expression profiling; MAPK: Mitogen-activated protein kinase; DAVID: Database for Annotation, Visualization and Integrated Discovery; TRIB1: Tribbles homolog 1; STK17: *B*serine/threonine kinase 17b; PTK2B: PTK2B protein tyrosine kinase 2 beta; C5AR1: Complement component 5a receptor 1; ZFP36: Zinc finger protein 36, C3H type, homolog; qRT-PCR: Quantitative real-time polymerase chain reaction experiments; IPA: Ingenuity Pathways Analysis; ERK1/2: Extracellular signal-regulated kinases; p38MAPK: p38 mitogen-activated protein kinase; JNK: c-Jun N-terminal kinase; AKT: RAC-alpha serine/threonine-protein kinase; PI3K: Phosphatidylinositol-3 kinase; MAPKK: Mitogen activated protein kinase kinase; C⁄EBPα: CCAAT⁄enhancer binding protein alpha; MEK1: Mitogen-activated ERK kinase 1; MKK4: MAP kinase kinase 4; hnRNP-E2: Poly(rC) binding protein 2.

## Competing interest

The authors declare that they have no competing interests.

## Authors’ contributions

FA, AZ, and LA contributed to the overall experimental design and wrote the manuscript. NC and GT conducted FISH experiments. PC and LI performed banding cytogenetics. ARR contributed to clinical data collection. AM and FCM contributed to molecular analysis experiments. FA and GS supervised the manuscript preparation and gave the final approval.

## Supplementary Material

Additional file 1: Table S1 Molecular cytogenetic characteristics of CML cases with variant t(9;22) translocations. Click here for file

Additional file 2 Supplementary Materials and Methods. Click here for file

Additional file 3: Table S2 Differentially expressed genes between CML cases with variant and classic t(9;22). Genes are rank-ordered according to fold change value. Click here for file

## References

[B1] Goldman JM Melo JV Chronic myeloid leukemia–advances in biology and new approaches to treatment N Engl J Med 2003 349 1451 1464 10.1056/NEJMra020777 14534339

[B2] Melo JV Barnes DJ Chronic myeloid leukemia as a model of disease evolution in human cancer Nat Rev Cancer 2007 7 441 453 10.1038/nrc2147 17522713

[B3] Mitelman F Johansson B Mertens F Mitelman Database of Chromosome Aberrations and Gene Fusions in Cancer 2012 http://www.cgap.nci.nih.gov/Chromosomes/Mitelman

[B4] Zagaria A Anelli L Albano F Storlazzi CT Liso A Roberti MG Buquicchio C Liso V Rocchi M Specchia G A fluorescence in situ hybridization study of complex t(9;22) in two chronic myelocytic leukemia cases with a masked Philadelphia chromosome Cancer Genet Cytogenet 2004 150 81 85 10.1016/j.cancergencyto.2003.08.018 15041230

[B5] Landstrom AP Tefferi A Fluorescent in situ hybridization in the diagnosis, prognosis, and treatment monitoring of chronic myeloid leukemia Leuk Lymphoma 2006 47 397 402 10.1080/10428190500353133 16396761

[B6] Huntly BJ Reid AG Bench AJ Campbell LJ Telford N Shepherd P Szer J Prince HM Turner P Grace C Nacheva EP Green AR Deletions of the derivative chromosome 9 occur at the time of the Philadelphia translocation and provide a powerful and independent prognostic indicator in chronic myeloid leukemia Blood 2001 98 1732 1738 10.1182/blood.V98.6.1732 11535505

[B7] Albano F Anelli L Zagaria A Coccaro N Casieri P Rossi AR Vicari L Liso V Rocchi M Specchia G Non random distribution of genomic features in breakpoint regions involved in chronic myeloid leukemia cases with variant t(9;22) or additional chromosomal rearrangements Mol Cancer 2010 9 120 10.1186/1476-4598-9-120 20500819 PMC2887383

[B8] Marzocchi G Castagnetti F Luatti S Baldazzi C Stacchini M Gugliotta G Amabile M Specchia G Sessarego M Giussani U Valori L Discepoli G Montaldi A Santoro A Bonaldi L Giudici G Cianciulli AM Giacobbi F Palandri F Pane F Saglio G Martinelli G Baccarani M Rosti G Testoni N Gruppo Italiano Malattie EMatologiche dell'Adulto (GIMEMA) Working Party on Chronic Myeloid Leukemia. Variant Philadelphia translocations: molecular-cytogenetic characterization and prognostic influence on frontline imatinib therapy, a GIMEMA Working Party on CML analysis Blood 2011 117 6793 6800 10.1182/blood-2011-01-328294 21447834

[B9] Cilloni D Saglio G Molecular pathways: BCR-ABL Clin Cancer Res 2012 18 4 930 937 10.1158/1078-0432.CCR-10-1613 22156549

[B10] Yokoyama T Kanno Y Yamazaki Y Takahara T Miyata S Nakamura T Trib1 links the MEK1/ERK pathway in myeloid leukemogenesis Blood 2010 116 2768 2775 10.1182/blood-2009-10-246264 20610816

[B11] Dougherty CJ Kubasiak LA Frazier DP Li H Xiong WC Bishopric NH Webster KA Mitochondrial signals initiate the activation of c-Jun N-terminal kinase (JNK) by hypoxia-reoxygenation FASEB J 2004 18 1060 1070 10.1096/fj.04-1505com 15226266

[B12] Cao Q McIsaac SM Stadnyk AW Human colonic epithelial cells detect and respond to C5a via apically expressed C5aR through the ERK pathway Am J Physiol Cell Physiol 2012 302 C1731 C1740 10.1152/ajpcell.00213.2011 22496247

[B13] Villuendas R Steegmann JL Pollán M Tracey L Granda A Fernández-Ruiz E Casado LF Martínez J Martínez P Lombardía L Villalón L Odriozola J Piris MA Identification of genes involved in imatinib resistance in CML: a gene-expression profiling approach Leukemia 2006 20 1047 1054 10.1038/sj.leu.2404197 16598311

[B14] Radich JP Dai H Mao M Oehler V Schelter J Druker B Sawyers C Shah N Stock W Willman CL Friend S Linsley PS Gene expression changes associated with progression and response in chronic myeloid leukemia Proc Natl Acad Sci U S A 2006 103 2794 2799 10.1073/pnas.0510423103 16477019 PMC1413797

