# Supplementary material for: Gene expression profiling of chronic myeloid leukemia with variant t(9;22) reveals a different signature from cases with classic translocation
Source: Mol Cancer. 2013 May 4;12:36. doi: 10.1186/1476-4598-12-36 (PMC3658885; doi:10.1186/1476-4598-12-36)
Supplement: Additional file 1: Table S1 — Molecular cytogenetic characteristics of CML cases with variant t(9;22) translocations. [file 1476-4598-12-36-S1.doc]

**Supplementary Table 1.** Molecular cytogenetic characteristics of CML cases with variant t(9;22) translocations

| **Case** | **Variant t(9;22) rearrangement** | **deletions on chromosome 9** | **deletions on chromosome 22** | **deletions on the third derivative chromosome** |
| --- | --- | --- | --- | --- |
| #1 | t(6;9;22)(p21.31;q34;q11) | + | + | + |
| #2 | t(9;21;22)(q34;q22.13;q11) | + | + | + |
| #3 | t(6;9;22)(p12.3;q34;q11) | + | - | + |
| #4 | t(9;13;22)(q34;q14.12;q11) | + | + | + |
| #5 | t(3;9;22)(p21.31;q34;q11) | - | + | - |
| #6 | t(9;10;22)(q34;p11.22;q11) | + | + | - |
| #7 | t(9;11;22)(q34;q13.1;q11) | + | + | + |
| #8 | t(4;9;22)(q12;q34;q11) | + | + | + |
