# Supplementary material for: Gene expression profiling of chronic myeloid leukemia with variant t(9;22) reveals a different signature from cases with classic translocation
Source: Mol Cancer. 2013 May 4;12:36. doi: 10.1186/1476-4598-12-36 (PMC3658885; doi:10.1186/1476-4598-12-36)
Supplement: Additional file 2 — Supplementary Materials and Methods. [file 1476-4598-12-36-S2.doc]

**Supplementary Materials and Methods**

*Banding Cytogenetic and Molecular Cytogenetic analyses*

Banding cytogenetics of 24–48-h culture was performed on bone marrow (BM) cells at diagnosis by standard techniques and evaluated by Giemsa–Trypsin–Giemsa banding at about the 400-band level, according to the ISCN 2009. Twenty metaphases were analyzed for each case.

FISH experiments were performed on BM samples at the CML onset using bacterial artificial chromosome (BAC) probes selected according to the University of California Santa Cruz (UCSC) database (http://genome.ucsc.edu/index.html; February 2009 release). Chromosome preparations were hybridized in situ with probes labeled by nick translation. All analyzed BM samples employed in GEP analysis showed 100% cells bearing the Ph chromosome. Reiterative FISH experiments were performed to make a precise identification of chromosomal breakpoints on other chromosomes involved in variant t(9;22) and evaluate the occurrence of genomic microdeletions.

*Microarray studies*

Total white blood cells were isolated from BM samples by hypotonic red cell lysis, washed and transferred into a guanidinium isothiocyanate-containing buffer. Total RNA was extracted by CsCl gradient centrifugation; RNA concentration and quality was determined using the NanoDrop ND-1000 (NanoDrop Technologies; http://www.nanodrop.com) and the 2100 Bioanalyzer (Agilent Technologies; http://www.agilent.com).Only cellular samples of high quality characterized by RNA integrity numbers ranging from 8 to 10 were selected for microarrays experiments. Twenty-five nanograms of each sample were labeled using the Low Input Quick Amp Labeling kit (Agilent Technologies). The specific gene chips used were Agilent SurePrint G3 Human GE 8x60K Microarray slide (Agilent Technologies), a new microarray format based on updated transcriptome databases for 27,958 Entrez Gene RNA targets and 7,419 additional probes for lincRNAs (long
intergenic non-coding RNAs). Scanned images were processed using the Agilent Feature Extraction software (v10.7.3.1), raw data were quantile normalized and differentially expressed genes were selected by unpaired t-test and multiple testing correction of Benjamini-Hochberg, considering as statistically significant those presenting a p-value less than 0.05 and a fold-change equal or higher than 2. Microarray data were analyzed using two different gene annotation databases (Database for Annotation, Visualization and Integrated Discovery, DAVID; Ingenuity Pathways Analysis, IPA) in order to determine whether the set of differentially expressed genes are involved in common processes, pathways, and underlying biological themes. The DAVID database (http://david.abcc.ncifcrf.gov/) performs a Gene-Enrichment and Functional Annotation Analysis to highlight the most relevant Gene Ontology terms associated with a given gene list. IPA software (www.ingenuity.com) assembles the genes on the basis of their direct or indirect interactions producing biological networks. Raw data are reported in the National Center for Biotechnology Information’s Gene Expression Omnibus database (assigned GEO accession number GSE41375).

*Quantitative real-time polymerase chain reaction*

To validate microarray data, quantitative real-time polymerase chain reaction experiments (qRT-PCR) (LightCycler 480II, Roche) were performed for all samples using the LightCycler 480 SYBR Green I Master mix and specific primer pairs selected according to the Primer3 software (http://frodo.wi.mit.edu). To standardize the quantification of each target gene, β-glucuronidase (*β-GUS*) and importin 8 (*IPO8*) genes served as endogenous controls. To validate the individual primer sets efficiency, linearity of amplification was studied over a broad concentration range using dilution series of a normal control cDNA.
